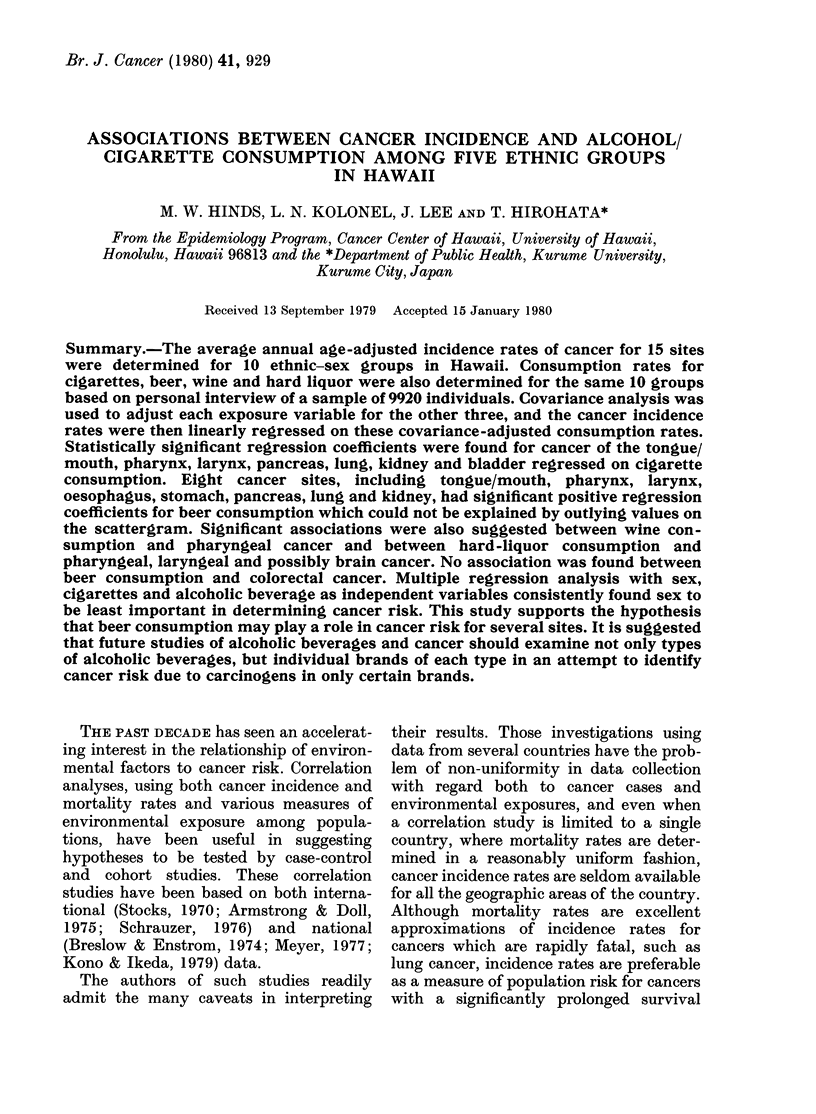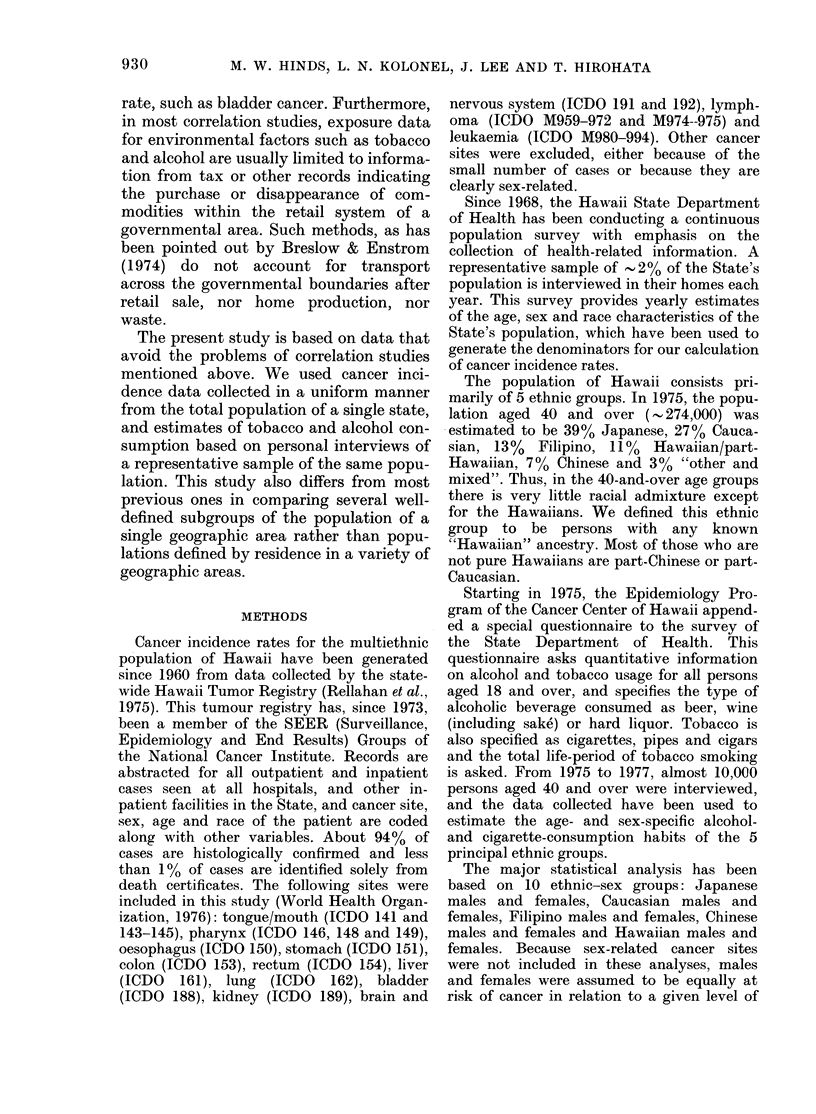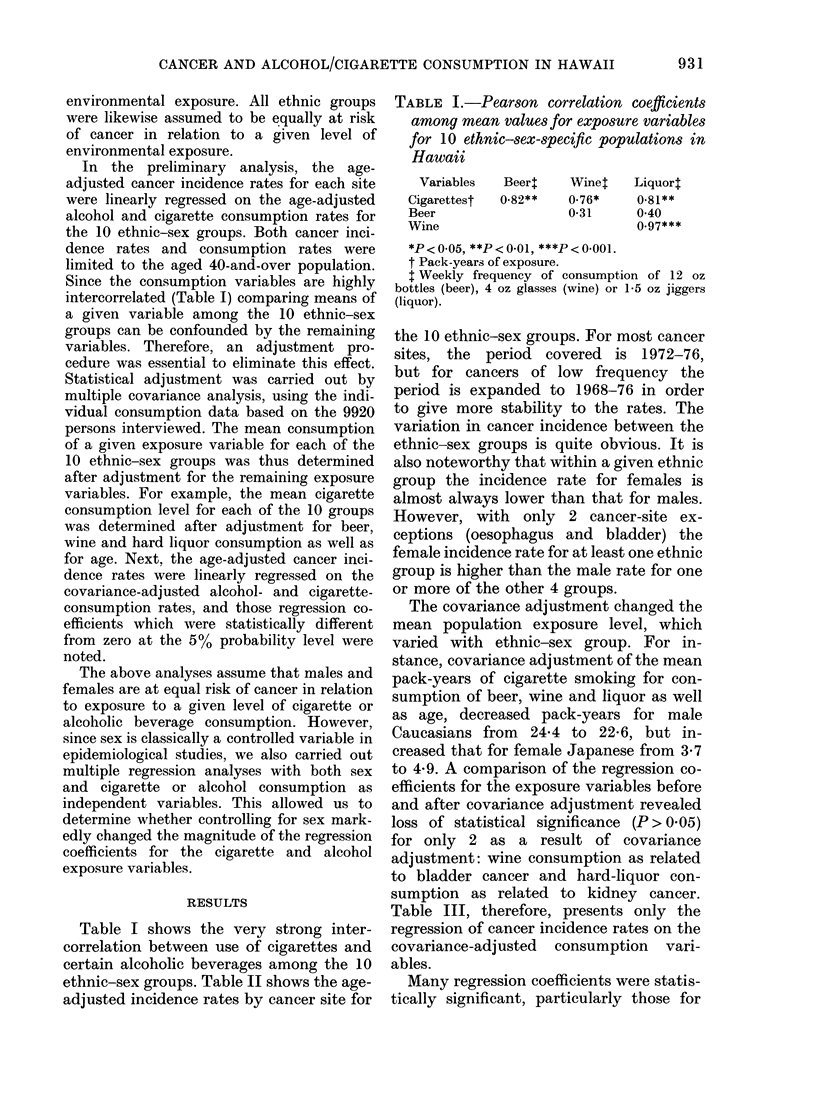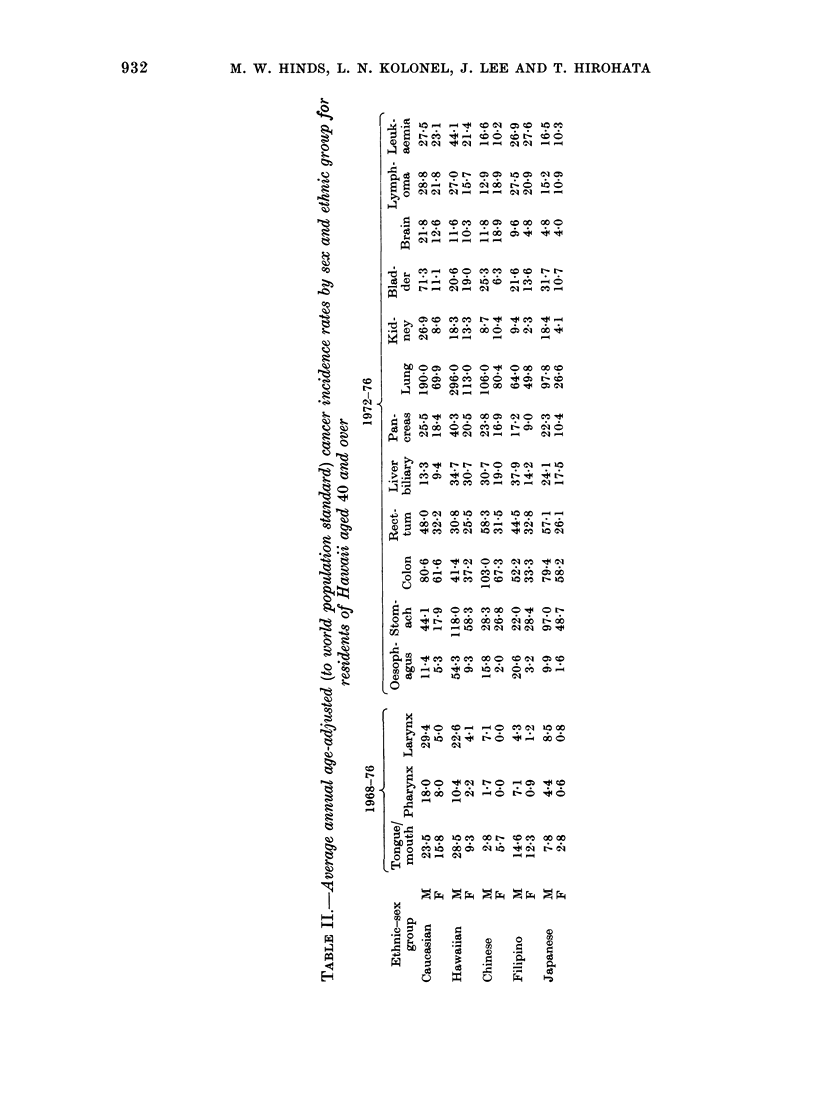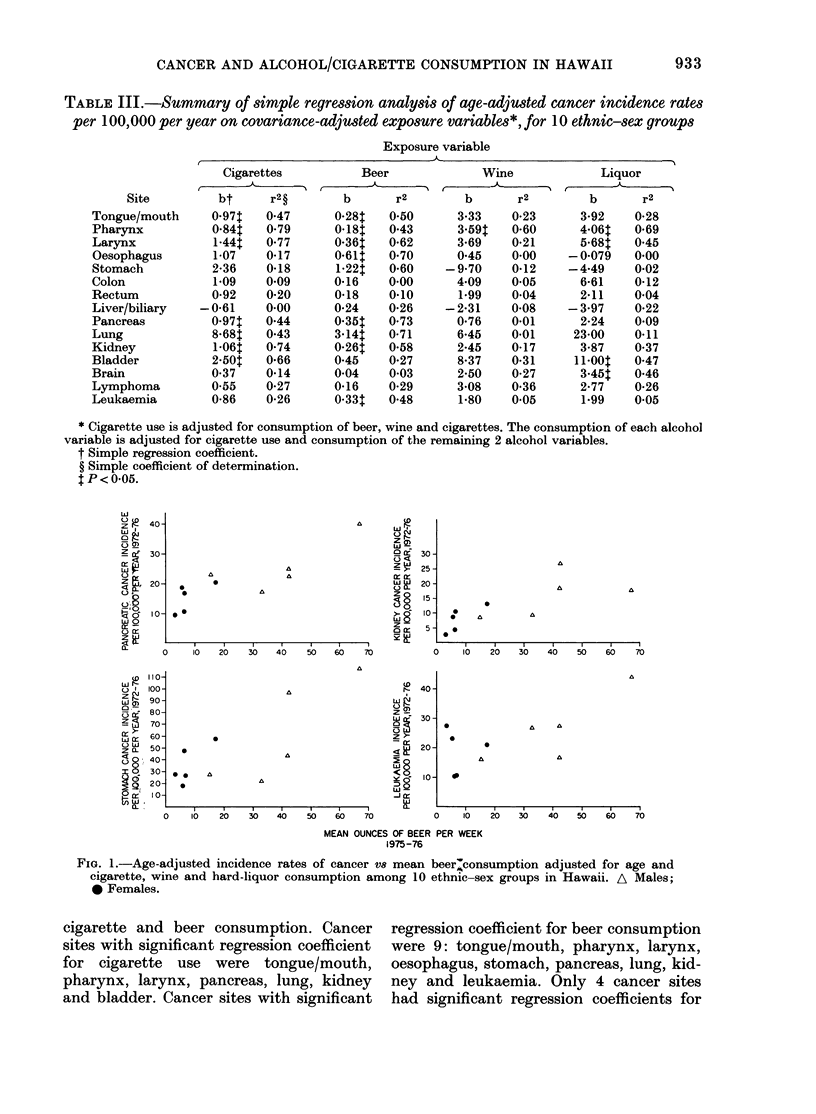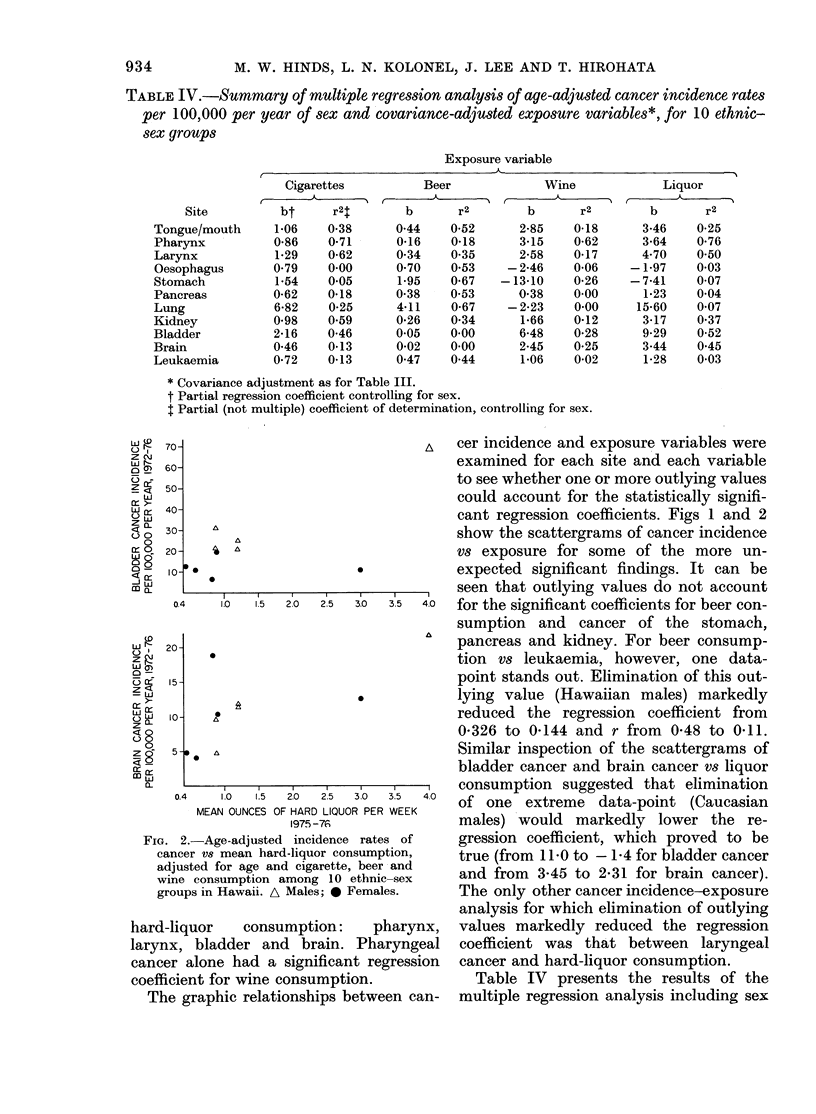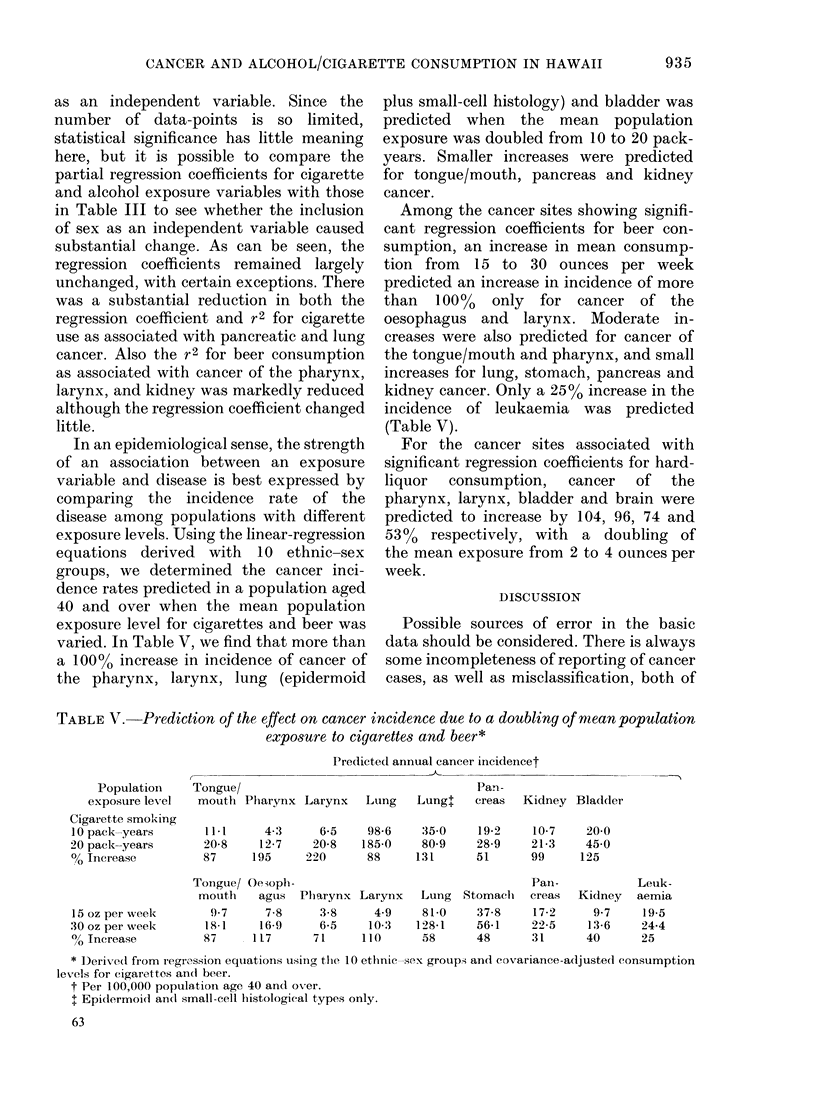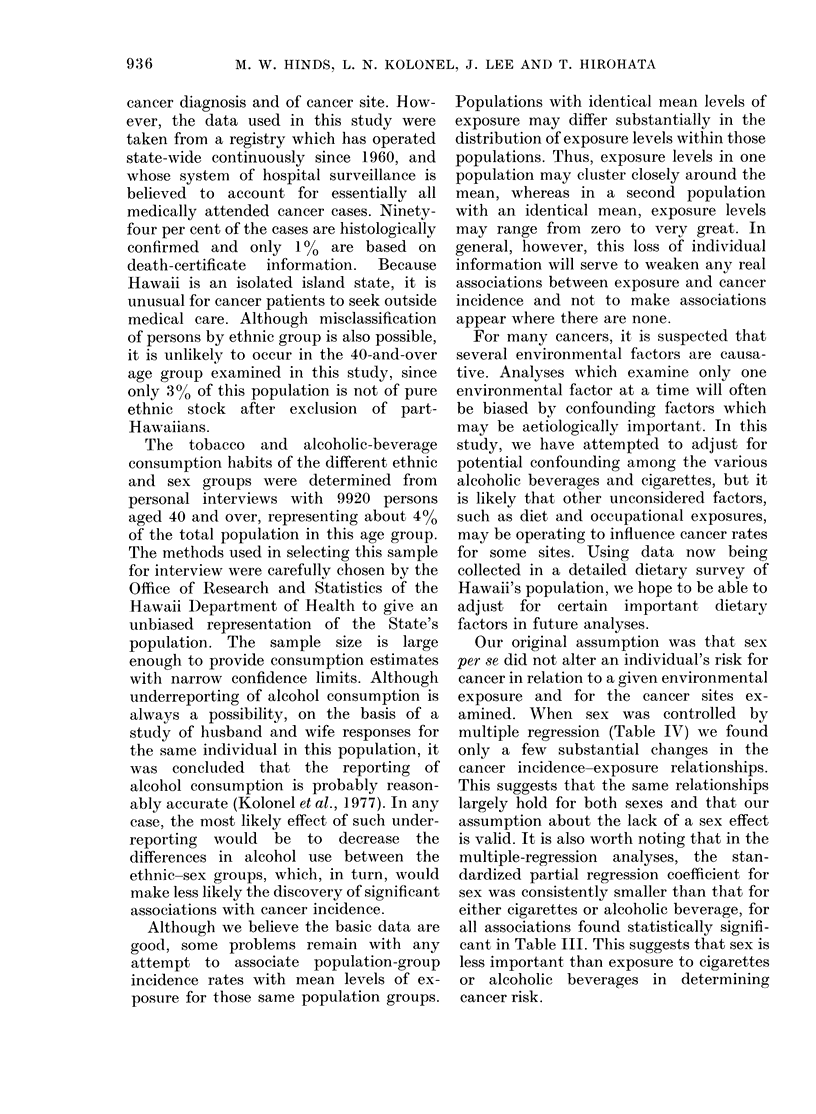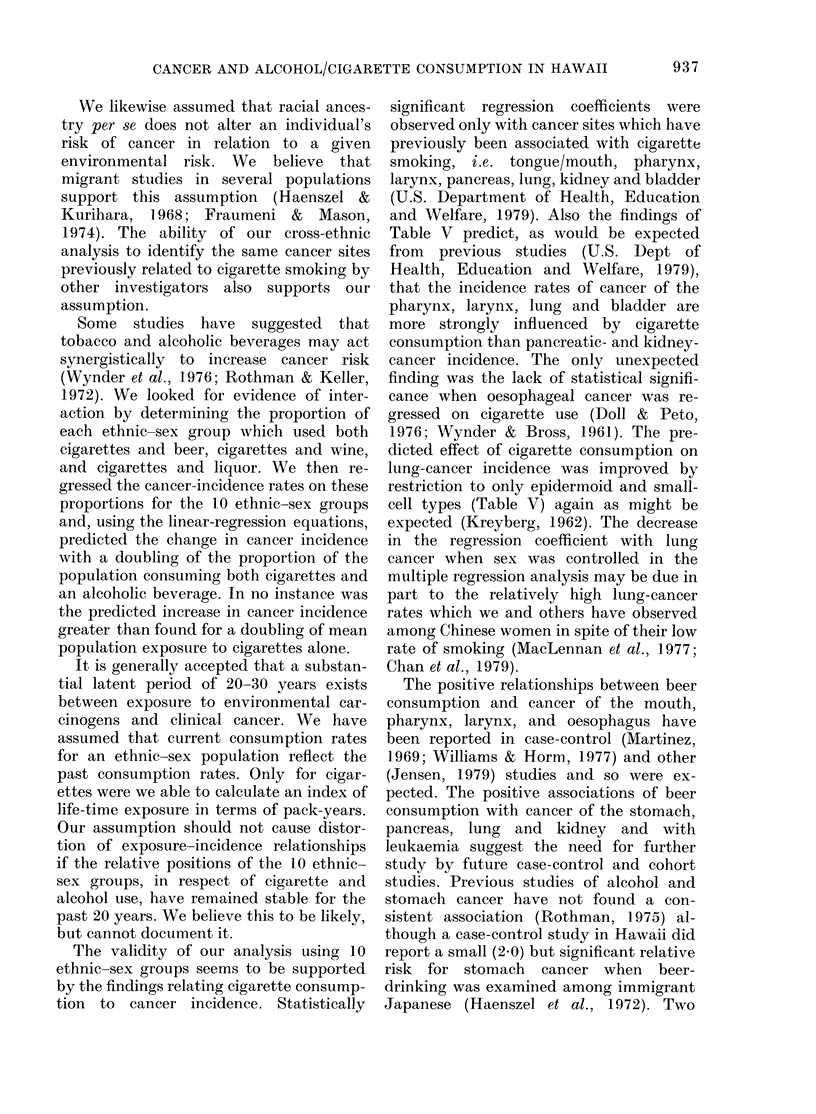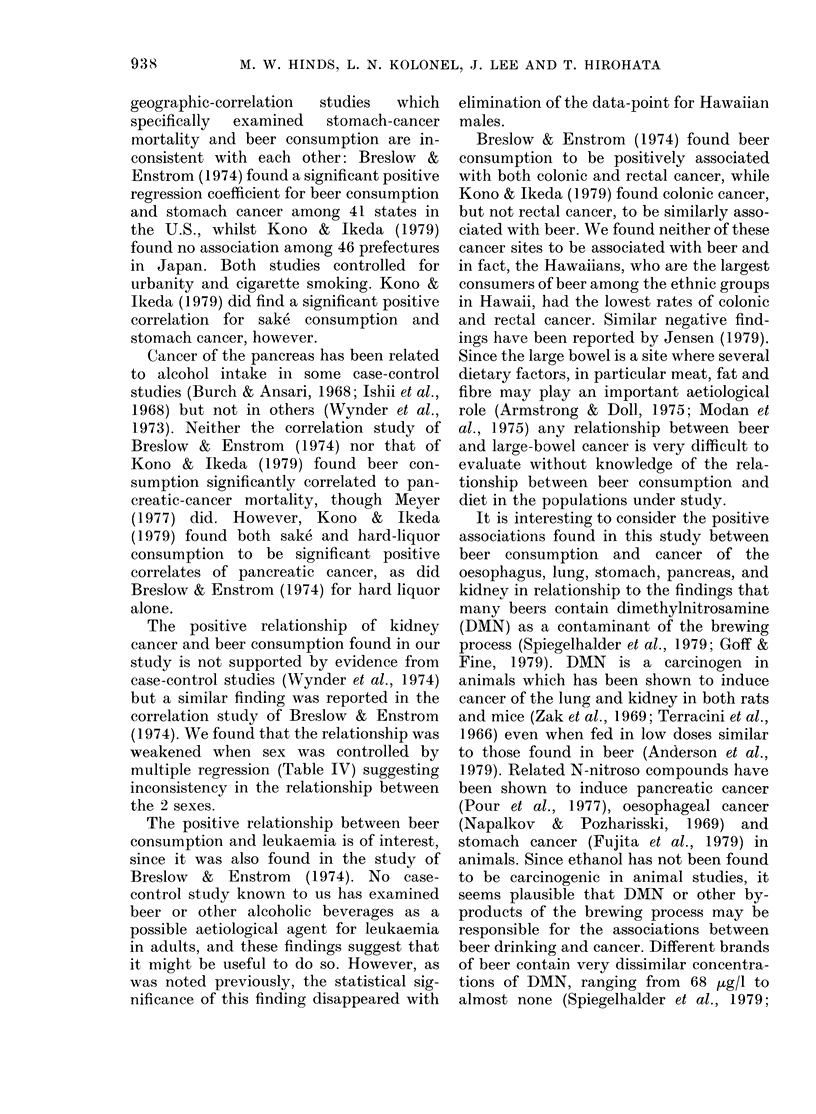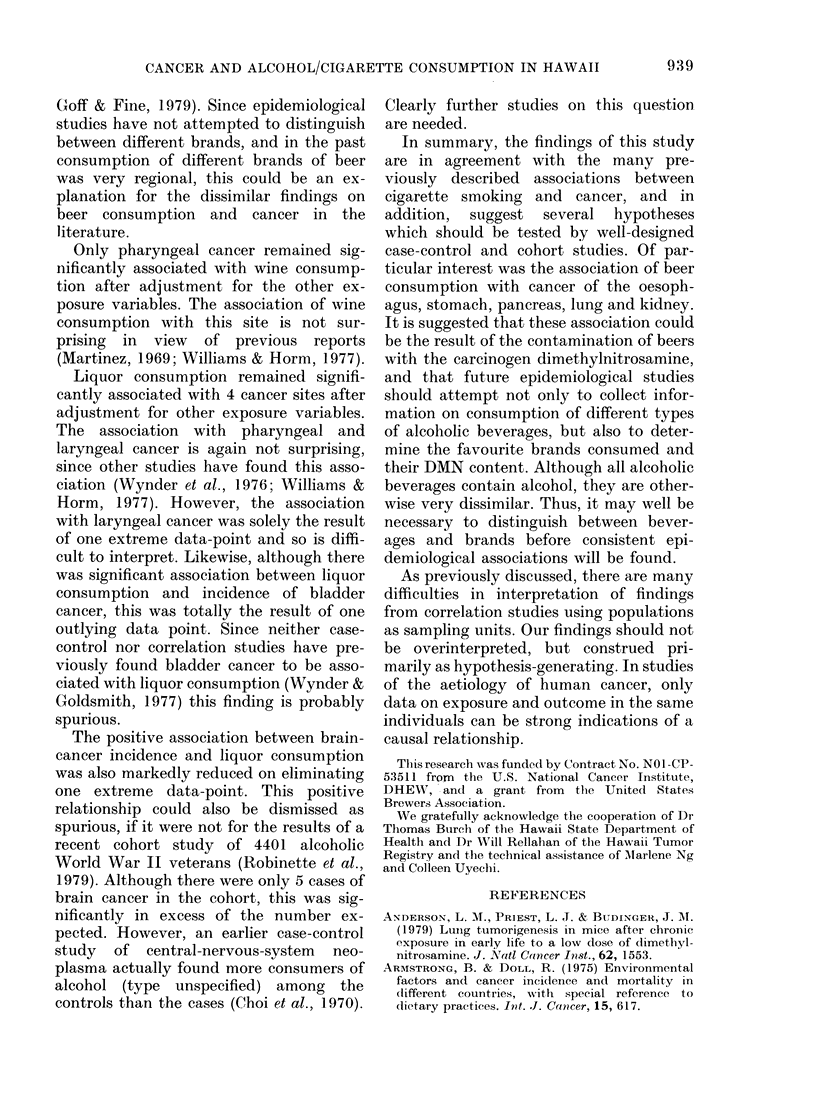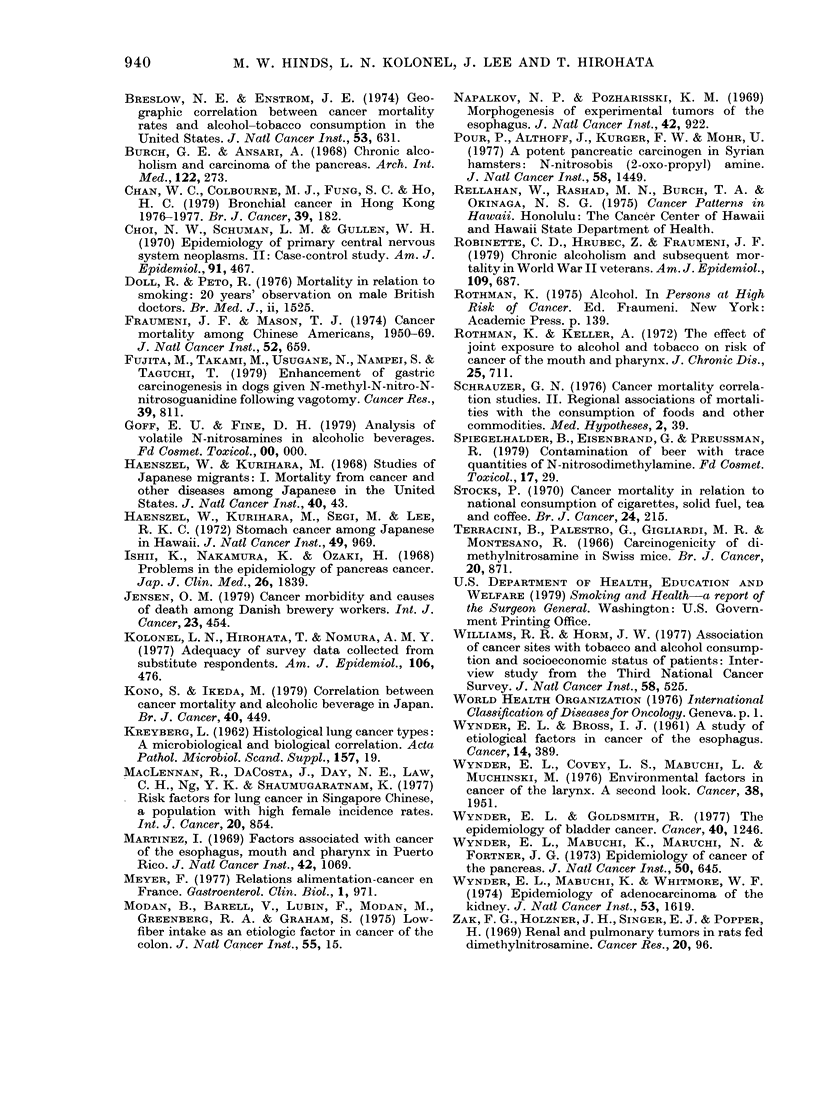# Associations between cancer incidence and alcohol/cigarette consumption among five ethnic groups in Hawaii.

**DOI:** 10.1038/bjc.1980.171

**Published:** 1980-06

**Authors:** M. W. Hinds, L. N. Kolonel, J. Lee, T. Hirohata

## Abstract

The average annual age-adjusted incidence rates of cancer for 15 sites were determined for 10 ethnic-sex groups in Hawaii. Consumption rates for cigarettes, beer, wine and hard liquor were also determined for the same 10 groups based on personal interview of a sample of 9920 individuals. Covariance analysis was used to adjust each exposure variable for the other three, and the cancer incidence rates were then linearly regressed on these covariance-adjusted consumption rates. Statistically significant regression coefficients were found for cancer of the tongue/mouth, pharynx, larynx, pancreas, lung, kidney and bladder regressed on cigarette consumption. Eight cancer sites, including tongue/mouth, pharynx, larynx, oesophagus, stomach, pancreas, lung and kidney, had significant positive regression coefficients for beer consumption which could not be explained by outlying values on the scattergram. Significant associations were also suggested between wine consumption and pharyngeal cancer and between hard-liquor consumption and pharyngeal, laryngeal and possibly brain cancer. No association was found between beer consumption and colorectal cancer. Multiple regression analysis with sex, cigarettes and alcoholic beverage as independent variables consistently found sex to be least important in determining cancer risk. This study supports the hypothesis that beer consumption may play a role in cancer risk for several sites. It is suggested that future studies of alcoholic beverages and cancer should examine not only types of alcoholic beverages, but individual brands of each type in an attempt to identify cancer risk due to carcinogens in only certain brands.


*Br. J. Cancer* (1980) **41**, 929

### ASSOCIATIONS BETWEEN CANCER INCIDENCE AND ALCOHOL/CIGARETTE CONSUMPTION AMONG FIVE ETHNIC GROUPS IN HAWAII

M. W. HINDS, L. N. KOLONEL, J. LEE AND T. HIROHATA*

*From the Epidemiology Program, Cancer Center of Hawaii, University of Hawaii, Honolulu, Hawaii* 96813 *and the *Department of Public Health, Kurume University, Kurume City, Japan*

Received 13 September 1979 Accepted 15 January 1980

**Summary.—The average annual age-adjusted incidence rates of cancer for 15 sites were determined for 10 ethnic–sex groups in Hawaii. Consumption rates for cigarettes, beer, wine and hard liquor were also determined for the same 10 groups based on personal interview of a sample of 9920 individuals. Covariance analysis was used to adjust each exposure variable for the other three, and the cancer incidence rates were then linearly regressed on these covariance-adjusted consumption rates. Statistically significant regression coefficients were found for cancer of the tongue/mouth, pharynx, larynx, pancreas, lung, kidney and bladder regressed on cigarette consumption. Eight cancer sites, including tongue/mouth, pharynx, larynx, oesophagus, stomach, pancreas, lung and kidney, had significant positive regression coefficients for beer consumption which could not be explained by outlying values on the scattergram. Significant associations were also suggested between wine consumption and pharyngeal cancer and between hard-liquor consumption and pharyngeal, laryngeal and possibly brain cancer. No association was found between beer consumption and colorectal cancer. Multiple regression analysis with sex, cigarettes and alcoholic beverage as independent variables consistently found sex to be least important in determining cancer risk. This study supports the hypothesis that beer consumption may play a role in cancer risk for several sites. It is suggested that future studies of alcoholic beverages and cancer should examine not only types of alcoholic beverages, but individual brands of each type in an attempt to identify cancer risk due to carcinogens in only certain brands.**

THE PAST DECADE has seen an accelerating interest in the relationship of environmental factors to cancer risk. Correlation analyses, using both cancer incidence and mortality rates and various measures of environmental exposure among populations, have been useful in suggesting hypotheses to be tested by case-control and cohort studies. These correlation studies have been based on both international (Stocks, 1970; Armstrong & Doll, 1975; Schrauzer, 1976) and national (Breslow & Enstrom, 1974; Meyer, 1977; Kono & Ikeda, 1979) data.

The authors of such studies readily admit the many caveats in interpreting their results. Those investigations using data from several countries have the problem of non-uniformity in data collection with regard both to cancer cases and environmental exposures, and even when a correlation study is limited to a single country, where mortality rates are determined in a reasonably uniform fashion, cancer incidence rates are seldom available for all the geographic areas of the country. Although mortality rates are excellent approximations of incidence rates for cancers which are rapidly fatal, such as lung cancer, incidence rates are preferable as a measure of population risk for cancers with a significantly prolonged survival rate, such as bladder cancer. Furthermore, in most correlation studies, exposure data for environmental factors such as tobacco and alcohol are usually limited to information from tax or other records indicating the purchase or disappearance of commodities within the retail system of a governmental area. Such methods, as has been pointed out by Breslow & Enstrom (1974) do not account for transport across the governmental boundaries after retail sale, nor home production, nor waste.

The present study is based on data that avoid the problems of correlation studies mentioned above. We used cancer incidence data collected in a uniform manner from the total population of a single state, and estimates of tobacco and alcohol consumption based on personal interviews of a representative sample of the same population. This study also differs from most previous ones in comparing several well-defined subgroups of the population of a single geographic area rather than populations defined by residence in a variety of geographic areas.

### METHODS

Cancer incidence rates for the multiethnic population of Hawaii have been generated since 1960 from data collected by the statewide Hawaii Tumor Registry (Rellahan *et al.*, 1975). This tumour registry has, since 1973, been a member of the SEER (Surveillance, Epidemiology and End Results) Groups of the National Cancer Institute. Records are abstracted for all outpatient and inpatient cases seen at all hospitals, and other inpatient facilities in the State, and cancer site, sex, age and race of the patient are coded along with other variables. About 94% of cases are histologically confirmed and less than 1% of cases are identified solely from death certificates. The following sites were included in this study (World Health Organization, 1976): tongue/mouth (ICDO 141 and 143–145), pharynx (ICDO 146, 148 and 149), oesophagus (ICDO 150), stomach (ICDO 151), colon (ICDO 153), rectum (ICDO 154), liver (ICDO 161), lung (ICDO 162), bladder (ICDO 188), kidney (ICDO 189), brain and nervous system (ICDO 191 and 192), lymphoma (ICDO M959–972 and M974–975) and leukaemia (ICDO M980–994). Other cancer sites were excluded, either because of the small number of cases or because they are clearly sex-related.

Since 1968, the Hawaii State Department of Health has been conducting a continuous population survey with emphasis on the collection of health-related information. A representative sample of ~2% of the State's population is interviewed in their homes each year. This survey provides yearly estimates of the age, sex and race characteristics of the State's population, which have been used to generate the denominators for our calculation of cancer incidence rates.

The population of Hawaii consists primarily of 5 ethnic groups. In 1975, the population aged 40 and over (~274,000) was estimated to be 39% Japanese, 27% Caucasian, 13% Filipino, 11% Hawaiian/part-Hawaiian, 7% Chinese and 3% "other and mixed". Thus, in the 40-and-over age groups there is very little racial admixture except for the Hawaiians. We defined this ethnic group to be persons with any known "Hawaiian" ancestry. Most of those who are not pure Hawaiians are part-Chinese or part-Caucasian.

Starting in 1975, the Epidemiology Program of the Cancer Center of Hawaii appended a special questionnaire to the survey of the State Department of Health. This questionnaire asks quantitative information on alcohol and tobacco usage for all persons aged 18 and over, and specifies the type of alcoholic beverage consumed as beer, wine (including saké) or hard liquor. Tobacco is also specified as cigarettes, pipes and cigars and the total life-period of tobacco smoking is asked. From 1975 to 1977, almost 10,000 persons aged 40 and over were interviewed, and the data collected have been used to estimate the age- and sex-specific alcohol- and cigarette-consumption habits of the 5 principal ethnic groups.

The major statistical analysis has been based on 10 ethnic–sex groups: Japanese males and females, Caucasian males and females, Filipino males and females, Chinese males and females and Hawaiian males and females. Because sex-related cancer sites were not included in these analyses, males and females were assumed to be equally at risk of cancer in relation to a given level of environmental exposure. All ethnic groups were likewise assumed to be equally at risk of cancer in relation to a given level of environmental exposure.

In the preliminary analysis, the age-adjusted cancer incidence rates for each site were linearly regressed on the age-adjusted alcohol and cigarette consumption rates for the 10 ethnic-sex groups. Both cancer incidence rates and consumption rates were limited to the aged 40-and-over population. Since the consumption variables are highly intercorrelated (Table I) comparing means of a given variable among the 10 ethnic–sex groups can be confounded by the remaining variables. Therefore, an adjustment procedure was essential to eliminate this effect. Statistical adjustment was carried out by multiple covariance analysis, using the individual consumption data based on the 9920 persons interviewed. The mean consumption of a given exposure variable for each of the 10 ethnic–sex groups was thus determined after adjustment for the remaining exposure variables. For example, the mean cigarette consumption level for each of the 10 groups was determined after adjustment for beer, wine and hard liquor consumption as well as for age. Next, the age-adjusted cancer incidence rates were linearly regressed on the covariance-adjusted alcohol- and cigarette-consumption rates, and those regression coefficients which were statistically different from zero at the 5% probability level were noted.

The above analyses assume that males and females are at equal risk of cancer in relation to exposure to a given level of cigarette or alcoholic beverage consumption. However, since sex is classically a controlled variable in epidemiological studies, we also carried out multiple regression analyses with both sex and cigarette or alcohol consumption as independent variables. This allowed us to determine whether controlling for sex markedly changed the magnitude of the regression coefficients for the cigarette and alcohol exposure variables.

### RESULTS

Table I shows the very strong intercorrelation between use of cigarettes and certain alcoholic beverages among the 10 ethnic–sex groups. Table II shows the age-adjusted incidence rates by cancer site for the 10 ethnic–sex groups. For most cancer sites, the period covered is 1972–76, but for cancers of low frequency the period is expanded to 1968–76 in order to give more stability to the rates. The variation in cancer incidence between the ethnic–sex groups is quite obvious. It is also noteworthy that within a given ethnic group the incidence rate for females is almost always lower than that for males. However, with only 2 cancer-site exceptions (oesophagus and bladder) the female incidence rate for at least one ethnic group is higher than the male rate for one or more of the other 4 groups.

TABLE I.—*Pearson correlation coefficients among mean values for exposure variables for 10 ethnic–sex-specific populations in Hawaii*

| Variables | Beer‡ | Wine‡ | Liquor‡ |
|---|---|---|---|
| Cigarettes† | 0·82** | 0·76* | 0·81** |
| Beer | | 0·31 | 0·40 |
| Wine | | | 0·97*** |

$^{*}P<0{\cdot}05$, $^{**}P<0{\cdot}01$, $^{***}P<0{\cdot}001$.

† Pack-years of exposure.

‡ Weekly frequency of consumption of 12 oz bottles (beer), 4 oz glasses (wine) or 1·5 oz jiggers (liquor).

The covariance adjustment changed the mean population exposure level, which varied with ethnic–sex group. For instance, covariance adjustment of the mean pack-years of cigarette smoking for consumption of beer, wine and liquor as well as age, decreased pack-years for male Caucasians from 24·4 to 22·6, but increased that for female Japanese from 3·7 to 4·9. A comparison of the regression coefficients for the exposure variables before and after covariance adjustment revealed loss of statistical significance ($P>0{\cdot}05$) for only 2 as a result of covariance adjustment: wine consumption as related to bladder cancer and hard-liquor consumption as related to kidney cancer. Table III, therefore, presents only the regression of cancer incidence rates on the covariance-adjusted consumption variables.

Many regression coefficients were statistically significant, particularly those for

TABLE II.—*Average annual age-adjusted (to world population standard) cancer incidence rates by sex and ethnic group for residents of Hawaii aged* 40 *and over*

| Ethnic–sex group | | 1968–76 | | | 1972–76 | | | | | | | | | | | |
|---|---|---|---|---|---|---|---|---|---|---|---|---|---|---|---|---|
| | | Tongue/ mouth | Pharynx | Larynx | Oesoph- agus | Stom- ach | Colon | Rect- tum | Liver biliary | Pan- creas | Lung | Kid- ney | Blad- der | Brain | Lymph- oma | Leuk- aemia |
| Caucasian | M | 23·5 | 18·0 | 29·4 | 11·4 | 44·1 | 80·6 | 48·0 | 13·3 | 25·5 | 190·0 | 26·9 | 71·3 | 21·8 | 28·8 | 27·5 |
| | F | 15·8 | 8·0 | 5·0 | 5·3 | 17·9 | 61·6 | 32·2 | 9·4 | 18·4 | 69·9 | 8·6 | 11·1 | 12·6 | 21·8 | 23·1 |
| Hawaiian | M | 28·5 | 10·4 | 22·6 | 54·3 | 118·0 | 41·4 | 30·8 | 34·7 | 40·3 | 296·0 | 18·3 | 20·6 | 11·6 | 27·0 | 44·1 |
| | F | 9·3 | 2·2 | 4·1 | 9·3 | 58·3 | 37·2 | 25·5 | 30·7 | 20·5 | 113·0 | 13·3 | 19·0 | 10·3 | 15·7 | 21·4 |
| Chinese | M | 2·8 | 1·7 | 7·1 | 15·8 | 28·3 | 103·0 | 58·3 | 30·7 | 23·8 | 106·0 | 8·7 | 25·3 | 11·8 | 12·9 | 16·6 |
| | F | 5·7 | 0·0 | 0·0 | 2·0 | 26·8 | 67·3 | 31·5 | 19·0 | 16·9 | 80·4 | 10·4 | 6·3 | 18·9 | 18·9 | 10·2 |
| Filipino | M | 14·6 | 7·1 | 4·3 | 20·6 | 22·0 | 52·2 | 44·5 | 37·9 | 17·2 | 64·0 | 9·4 | 21·6 | 9·6 | 27·5 | 26·9 |
| | F | 12·3 | 0·9 | 1·2 | 3·2 | 28·4 | 33·3 | 32·8 | 14·2 | 9·0 | 49·8 | 2·3 | 13·6 | 4·8 | 20·9 | 27·6 |
| Japanese | M | 7·8 | 4·4 | 8·5 | 9·9 | 97·0 | 79·4 | 57·1 | 24·1 | 22·3 | 97·8 | 18·4 | 31·7 | 4·8 | 15·2 | 16·5 |
| | F | 2·8 | 0·6 | 0·8 | 1·6 | 48·7 | 58·2 | 26·1 | 17·5 | 10·4 | 26·6 | 4·1 | 10·7 | 4·0 | 10·9 | 10·3 |

TABLE III.—*Summary of simple regression analysis of age-adjusted cancer incidence rates per* 100,000 *per year on covariance-adjusted exposure variables*, for* 10 *ethnic–sex groups*

| | Exposure variable | | | | | | | |
|---|---|---|---|---|---|---|---|---|
| | Cigarettes | | Beer | | Wine | | Liquor | |
| Site | b† | $r^2$§ | b | $r^2$ | b | $r^2$ | b | $r^2$ |
| Tongue/mouth | 0·97‡ | 0·47 | 0·28‡ | 0·50 | 3·33 | 0·23 | 3·92 | 0·28 |
| Pharynx | 0·84‡ | 0·79 | 0·18‡ | 0·43 | 3·59‡ | 0·60 | 4·06‡ | 0·69 |
| Larynx | 1·44‡ | 0·77 | 0·36‡ | 0·62 | 3·69 | 0·21 | 5·68‡ | 0·45 |
| Oesophagus | 1·07 | 0·17 | 0·61‡ | 0·70 | 0·45 | 0·00 | −0·079 | 0·00 |
| Stomach | 2·36 | 0·18 | 1·22‡ | 0·60 | −9·70 | 0·12 | −4·49 | 0·02 |
| Colon | 1·09 | 0·09 | 0·16 | 0·00 | 4·09 | 0·05 | 6·61 | 0·12 |
| Rectum | 0·92 | 0·20 | 0·18 | 0·10 | 1·99 | 0·04 | 2·11 | 0·04 |
| Liver/biliary | −0·61 | 0·00 | 0·24 | 0·26 | −2·31 | 0·08 | −3·97 | 0·22 |
| Pancreas | 0·97‡ | 0·44 | 0·35‡ | 0·73 | 0·76 | 0·01 | 2·24 | 0·09 |
| Lung | 8·68‡ | 0·43 | 3·14‡ | 0·71 | 6·45 | 0·01 | 23·00 | 0·11 |
| Kidney | 1·06‡ | 0·74 | 0·26‡ | 0·58 | 2·45 | 0·17 | 3·87 | 0·37 |
| Bladder | 2·50‡ | 0·66 | 0·45 | 0·27 | 8·37 | 0·31 | 11·00‡ | 0·47 |
| Brain | 0·37 | 0·14 | 0·04 | 0·03 | 2·50 | 0·27 | 3·45‡ | 0·46 |
| Lymphoma | 0·55 | 0·27 | 0·16 | 0·29 | 3·08 | 0·36 | 2·77 | 0·26 |
| Leukaemia | 0·86 | 0·26 | 0·33‡ | 0·48 | 1·80 | 0·05 | 1·99 | 0·05 |

* Cigarette use is adjusted for consumption of beer, wine and cigarettes. The consumption of each alcohol variable is adjusted for cigarette use and consumption of the remaining 2 alcohol variables.
† Simple regression coefficient.
§ Simple coefficient of determination.
‡ $P < 0·05$.

FIG. 1.—Age-adjusted incidence rates of cancer *vs* mean beer consumption adjusted for age and cigarette, wine and hard-liquor consumption among 10 ethnic–sex groups in Hawaii. △ Males; ● Females.

cigarette and beer consumption. Cancer sites with significant regression coefficient for cigarette use were tongue/mouth, pharynx, larynx, pancreas, lung, kidney and bladder. Cancer sites with significant regression coefficient for beer consumption were 9: tongue/mouth, pharynx, larynx, oesophagus, stomach, pancreas, lung, kidney and leukaemia. Only 4 cancer sites had significant regression coefficients for

TABLE IV.—*Summary of multiple regression analysis of age-adjusted cancer incidence rates per* 100,000 *per year of sex and covariance-adjusted exposure variables*, for* 10 *ethnic–sex groups*

| | Exposure variable | | | | | | | |
|---|---|---|---|---|---|---|---|---|
| | Cigarettes | | Beer | | Wine | | Liquor | |
| Site | b† | $r^2$‡ | b | $r^2$ | b | $r^2$ | b | $r^2$ |
| Tongue/mouth | 1·06 | 0·38 | 0·44 | 0·52 | 2·85 | 0·18 | 3·46 | 0·25 |
| Pharynx | 0·86 | 0·71 | 0·16 | 0·18 | 3·15 | 0·62 | 3·64 | 0·76 |
| Larynx | 1·29 | 0·62 | 0·34 | 0·35 | 2·58 | 0·17 | 4·70 | 0·50 |
| Oesophagus | 0·79 | 0·00 | 0·70 | 0·53 | −2·46 | 0·06 | −1·97 | 0·03 |
| Stomach | 1·54 | 0·05 | 1·95 | 0·67 | −13·10 | 0·26 | −7·41 | 0·07 |
| Pancreas | 0·62 | 0·18 | 0·38 | 0·53 | 0·38 | 0·00 | 1·23 | 0·04 |
| Lung | 6·82 | 0·25 | 4·11 | 0·67 | −2·23 | 0·00 | 15·60 | 0·07 |
| Kidney | 0·98 | 0·59 | 0·26 | 0·34 | 1·66 | 0·12 | 3·17 | 0·37 |
| Bladder | 2·16 | 0·46 | 0·05 | 0·00 | 6·48 | 0·28 | 9·29 | 0·52 |
| Brain | 0·46 | 0·13 | 0·02 | 0·00 | 2·45 | 0·25 | 3·44 | 0·45 |
| Leukaemia | 0·72 | 0·13 | 0·47 | 0·44 | 1·06 | 0·02 | 1·28 | 0·03 |

* Covariance adjustment as for Table III.
† Partial regression coefficient controlling for sex.
‡ Partial (not multiple) coefficient of determination, controlling for sex.

FIG. 2.—Age-adjusted incidence rates of cancer *vs* mean hard-liquor consumption, adjusted for age and cigarette, beer and wine consumption among 10 ethnic–sex groups in Hawaii. △ Males; ● Females.

hard-liquor consumption: pharynx, larynx, bladder and brain. Pharyngeal cancer alone had a significant regression coefficient for wine consumption.

The graphic relationships between cancer incidence and exposure variables were examined for each site and each variable to see whether one or more outlying values could account for the statistically significant regression coefficients. Figs 1 and 2 show the scattergrams of cancer incidence *vs* exposure for some of the more unexpected significant findings. It can be seen that outlying values do not account for the significant coefficients for beer consumption and cancer of the stomach, pancreas and kidney. For beer consumption *vs* leukaemia, however, one data-point stands out. Elimination of this outlying value (Hawaiian males) markedly reduced the regression coefficient from 0·326 to 0·144 and *r* from 0·48 to 0·11. Similar inspection of the scattergrams of bladder cancer and brain cancer *vs* liquor consumption suggested that elimination of one extreme data-point (Caucasian males) would markedly lower the regression coefficient, which proved to be true (from 11·0 to −1·4 for bladder cancer and from 3·45 to 2·31 for brain cancer). The only other cancer incidence–exposure analysis for which elimination of outlying values markedly reduced the regression coefficient was that between laryngeal cancer and hard-liquor consumption.

Table IV presents the results of the multiple regression analysis including sex as an independent variable. Since the number of data-points is so limited, statistical significance has little meaning here, but it is possible to compare the partial regression coefficients for cigarette and alcohol exposure variables with those in Table III to see whether the inclusion of sex as an independent variable caused substantial change. As can be seen, the regression coefficients remained largely unchanged, with certain exceptions. There was a substantial reduction in both the regression coefficient and $r^2$ for cigarette use as associated with pancreatic and lung cancer. Also the $r^2$ for beer consumption as associated with cancer of the pharynx, larynx, and kidney was markedly reduced although the regression coefficient changed little.

In an epidemiological sense, the strength of an association between an exposure variable and disease is best expressed by comparing the incidence rate of the disease among populations with different exposure levels. Using the linear-regression equations derived with 10 ethnic–sex groups, we determined the cancer incidence rates predicted in a population aged 40 and over when the mean population exposure level for cigarettes and beer was varied. In Table V, we find that more than a 100% increase in incidence of cancer of the pharynx, larynx, lung (epidermoid plus small-cell histology) and bladder was predicted when the mean population exposure was doubled from 10 to 20 pack-years. Smaller increases were predicted for tongue/mouth, pancreas and kidney cancer.

Among the cancer sites showing significant regression coefficients for beer consumption, an increase in mean consumption from 15 to 30 ounces per week predicted an increase in incidence of more than 100% only for cancer of the oesophagus and larynx. Moderate increases were also predicted for cancer of the tongue/mouth and pharynx, and small increases for lung, stomach, pancreas and kidney cancer. Only a 25% increase in the incidence of leukaemia was predicted (Table V).

For the cancer sites associated with significant regression coefficients for hard-liquor consumption, cancer of the pharynx, larynx, bladder and brain were predicted to increase by 104, 96, 74 and 53% respectively, with a doubling of the mean exposure from 2 to 4 ounces per week.

### DISCUSSION

Possible sources of error in the basic data should be considered. There is always some incompleteness of reporting of cancer cases, as well as misclassification, both of

TABLE V.—*Prediction of the effect on cancer incidence due to a doubling of mean population exposure to cigarettes and beer**

| Population exposure level | Predicted annual cancer incidence† | | | | | | | |
|---|---|---|---|---|---|---|---|---|
| | Tongue/ mouth | Pharynx | Larynx | Lung | Lung‡ | Pan- creas | Kidney | Bladder |
| Cigarette smoking | | | | | | | | |
| 10 pack–years | 11·1 | 4·3 | 6·5 | 98·6 | 35·0 | 19·2 | 10·7 | 20·0 |
| 20 pack–years | 20·8 | 12·7 | 20·8 | 185·0 | 80·9 | 28·9 | 21·3 | 45·0 |
| % Increase | 87 | 195 | 220 | 88 | 131 | 51 | 99 | 125 |

| Population exposure level | Tongue/ mouth | Oesoph- agus | Pharynx | Larynx | Lung | Stomach | Pan- creas | Kidney | Leuk- aemia |
|---|---|---|---|---|---|---|---|---|---|
| 15 oz per week | 9·7 | 7·8 | 3·8 | 4·9 | 81·0 | 37·8 | 17·2 | 9·7 | 19·5 |
| 30 oz per week | 18·1 | 16·9 | 6·5 | 10·3 | 128·1 | 56·1 | 22·5 | 13·6 | 24·4 |
| % Increase | 87 | 117 | 71 | 110 | 58 | 48 | 31 | 40 | 25 |

* Derived from regression equations using the 10 ethnic–sex groups and covariance-adjusted consumption levels for cigarettes and beer.
† Per 100,000 population age 40 and over.
‡ Epidermoid and small-cell histological types only.

cancer diagnosis and of cancer site. However, the data used in this study were taken from a registry which has operated state-wide continuously since 1960, and whose system of hospital surveillance is believed to account for essentially all medically attended cancer cases. Ninety-four per cent of the cases are histologically confirmed and only 1% are based on death-certificate information. Because Hawaii is an isolated island state, it is unusual for cancer patients to seek outside medical care. Although misclassification of persons by ethnic group is also possible, it is unlikely to occur in the 40-and-over age group examined in this study, since only 3% of this population is not of pure ethnic stock after exclusion of part-Hawaiians.

The tobacco and alcoholic-beverage consumption habits of the different ethnic and sex groups were determined from personal interviews with 9920 persons aged 40 and over, representing about 4% of the total population in this age group. The methods used in selecting this sample for interview were carefully chosen by the Office of Research and Statistics of the Hawaii Department of Health to give an unbiased representation of the State's population. The sample size is large enough to provide consumption estimates with narrow confidence limits. Although underreporting of alcohol consumption is always a possibility, on the basis of a study of husband and wife responses for the same individual in this population, it was concluded that the reporting of alcohol consumption is probably reasonably accurate (Kolonel *et al.*, 1977). In any case, the most likely effect of such underreporting would be to decrease the differences in alcohol use between the ethnic–sex groups, which, in turn, would make less likely the discovery of significant associations with cancer incidence.

Although we believe the basic data are good, some problems remain with any attempt to associate population-group incidence rates with mean levels of exposure for those same population groups. Populations with identical mean levels of exposure may differ substantially in the distribution of exposure levels within those populations. Thus, exposure levels in one population may cluster closely around the mean, whereas in a second population with an identical mean, exposure levels may range from zero to very great. In general, however, this loss of individual information will serve to weaken any real associations between exposure and cancer incidence and not to make associations appear where there are none.

For many cancers, it is suspected that several environmental factors are causative. Analyses which examine only one environmental factor at a time will often be biased by confounding factors which may be aetiologically important. In this study, we have attempted to adjust for potential confounding among the various alcoholic beverages and cigarettes, but it is likely that other unconsidered factors, such as diet and occupational exposures, may be operating to influence cancer rates for some sites. Using data now being collected in a detailed dietary survey of Hawaii's population, we hope to be able to adjust for certain important dietary factors in future analyses.

Our original assumption was that sex *per se* did not alter an individual's risk for cancer in relation to a given environmental exposure and for the cancer sites examined. When sex was controlled by multiple regression (Table IV) we found only a few substantial changes in the cancer incidence–exposure relationships. This suggests that the same relationships largely hold for both sexes and that our assumption about the lack of a sex effect is valid. It is also worth noting that in the multiple-regression analyses, the standardized partial regression coefficient for sex was consistently smaller than that for either cigarettes or alcoholic beverage, for all associations found statistically significant in Table III. This suggests that sex is less important than exposure to cigarettes or alcoholic beverages in determining cancer risk.

We likewise assumed that racial ancestry *per se* does not alter an individual's risk of cancer in relation to a given environmental risk. We believe that migrant studies in several populations support this assumption (Haenszel & Kurihara, 1968; Fraumeni & Mason, 1974). The ability of our cross-ethnic analysis to identify the same cancer sites previously related to cigarette smoking by other investigators also supports our assumption.

Some studies have suggested that tobacco and alcoholic beverages may act synergistically to increase cancer risk (Wynder *et al.*, 1976; Rothman & Keller, 1972). We looked for evidence of interaction by determining the proportion of each ethnic–sex group which used both cigarettes and beer, cigarettes and wine, and cigarettes and liquor. We then regressed the cancer-incidence rates on these proportions for the 10 ethnic–sex groups and, using the linear-regression equations, predicted the change in cancer incidence with a doubling of the proportion of the population consuming both cigarettes and an alcoholic beverage. In no instance was the predicted increase in cancer incidence greater than found for a doubling of mean population exposure to cigarettes alone.

It is generally accepted that a substantial latent period of 20–30 years exists between exposure to environmental carcinogens and clinical cancer. We have assumed that current consumption rates for an ethnic–sex population reflect the past consumption rates. Only for cigarettes were we able to calculate an index of life-time exposure in terms of pack-years. Our assumption should not cause distortion of exposure–incidence relationships if the relative positions of the 10 ethnic–sex groups, in respect of cigarette and alcohol use, have remained stable for the past 20 years. We believe this to be likely, but cannot document it.

The validity of our analysis using 10 ethnic–sex groups seems to be supported by the findings relating cigarette consumption to cancer incidence. Statistically significant regression coefficients were observed only with cancer sites which have previously been associated with cigarette smoking, *i.e.* tongue/mouth, pharynx, larynx, pancreas, lung, kidney and bladder (U.S. Department of Health, Education and Welfare, 1979). Also the findings of Table V predict, as would be expected from previous studies (U.S. Dept of Health, Education and Welfare, 1979), that the incidence rates of cancer of the pharynx, larynx, lung and bladder are more strongly influenced by cigarette consumption than pancreatic- and kidney-cancer incidence. The only unexpected finding was the lack of statistical significance when oesophageal cancer was regressed on cigarette use (Doll & Peto, 1976; Wynder & Bross, 1961). The predicted effect of cigarette consumption on lung-cancer incidence was improved by restriction to only epidermoid and small-cell types (Table V) again as might be expected (Kreyberg, 1962). The decrease in the regression coefficient with lung cancer when sex was controlled in the multiple regression analysis may be due in part to the relatively high lung-cancer rates which we and others have observed among Chinese women in spite of their low rate of smoking (MacLennan *et al.*, 1977; Chan *et al.*, 1979).

The positive relationships between beer consumption and cancer of the mouth, pharynx, larynx, and oesophagus have been reported in case-control (Martinez, 1969; Williams & Horm, 1977) and other (Jensen, 1979) studies and so were expected. The positive associations of beer consumption with cancer of the stomach, pancreas, lung and kidney and with leukaemia suggest the need for further study by future case-control and cohort studies. Previous studies of alcohol and stomach cancer have not found a consistent association (Rothman, 1975) although a case-control study in Hawaii did report a small (2·0) but significant relative risk for stomach cancer when beer-drinking was examined among immigrant Japanese (Haenszel *et al.*, 1972). Two geographic-correlation studies which specifically examined stomach-cancer mortality and beer consumption are inconsistent with each other: Breslow & Enstrom (1974) found a significant positive regression coefficient for beer consumption and stomach cancer among 41 states in the U.S., whilst Kono & Ikeda (1979) found no association among 46 prefectures in Japan. Both studies controlled for urbanity and cigarette smoking. Kono & Ikeda (1979) did find a significant positive correlation for saké consumption and stomach cancer, however.

Cancer of the pancreas has been related to alcohol intake in some case-control studies (Burch & Ansari, 1968; Ishii *et al.*, 1968) but not in others (Wynder *et al.*, 1973). Neither the correlation study of Breslow & Enstrom (1974) nor that of Kono & Ikeda (1979) found beer consumption significantly correlated to pancreatic-cancer mortality, though Meyer (1977) did. However, Kono & Ikeda (1979) found both saké and hard-liquor consumption to be significant positive correlates of pancreatic cancer, as did Breslow & Enstrom (1974) for hard liquor alone.

The positive relationship of kidney cancer and beer consumption found in our study is not supported by evidence from case-control studies (Wynder *et al.*, 1974) but a similar finding was reported in the correlation study of Breslow & Enstrom (1974). We found that the relationship was weakened when sex was controlled by multiple regression (Table IV) suggesting inconsistency in the relationship between the 2 sexes.

The positive relationship between beer consumption and leukaemia is of interest, since it was also found in the study of Breslow & Enstrom (1974). No case-control study known to us has examined beer or other alcoholic beverages as a possible aetiological agent for leukaemia in adults, and these findings suggest that it might be useful to do so. However, as was noted previously, the statistical significance of this finding disappeared with elimination of the data-point for Hawaiian males.

Breslow & Enstrom (1974) found beer consumption to be positively associated with both colonic and rectal cancer, while Kono & Ikeda (1979) found colonic cancer, but not rectal cancer, to be similarly associated with beer. We found neither of these cancer sites to be associated with beer and in fact, the Hawaiians, who are the largest consumers of beer among the ethnic groups in Hawaii, had the lowest rates of colonic and rectal cancer. Similar negative findings have been reported by Jensen (1979). Since the large bowel is a site where several dietary factors, in particular meat, fat and fibre may play an important aetiological role (Armstrong & Doll, 1975; Modan *et al.*, 1975) any relationship between beer and large-bowel cancer is very difficult to evaluate without knowledge of the relationship between beer consumption and diet in the populations under study.

It is interesting to consider the positive associations found in this study between beer consumption and cancer of the oesophagus, lung, stomach, pancreas, and kidney in relationship to the findings that many beers contain dimethylnitrosamine (DMN) as a contaminant of the brewing process (Spiegelhalder *et al.*, 1979; Goff & Fine, 1979). DMN is a carcinogen in animals which has been shown to induce cancer of the lung and kidney in both rats and mice (Zak *et al.*, 1969; Terracini *et al.*, 1966) even when fed in low doses similar to those found in beer (Anderson *et al.*, 1979). Related N-nitroso compounds have been shown to induce pancreatic cancer (Pour *et al.*, 1977), oesophageal cancer (Napalkov & Pozharisski, 1969) and stomach cancer (Fujita *et al.*, 1979) in animals. Since ethanol has not been found to be carcinogenic in animal studies, it seems plausible that DMN or other by-products of the brewing process may be responsible for the associations between beer drinking and cancer. Different brands of beer contain very dissimilar concentrations of DMN, ranging from 68 $\mu$g/l to almost none (Spiegelhalder *et al.*, 1979;

Goff & Fine, 1979). Since epidemiological studies have not attempted to distinguish between different brands, and in the past consumption of different brands of beer was very regional, this could be an explanation for the dissimilar findings on beer consumption and cancer in the literature.

Only pharyngeal cancer remained significantly associated with wine consumption after adjustment for the other exposure variables. The association of wine consumption with this site is not surprising in view of previous reports (Martinez, 1969; Williams & Horm, 1977).

Liquor consumption remained significantly associated with 4 cancer sites after adjustment for other exposure variables. The association with pharyngeal and laryngeal cancer is again not surprising, since other studies have found this association (Wynder *et al.*, 1976; Williams & Horm, 1977). However, the association with laryngeal cancer was solely the result of one extreme data-point and so is difficult to interpret. Likewise, although there was significant association between liquor consumption and incidence of bladder cancer, this was totally the result of one outlying data point. Since neither case-control nor correlation studies have previously found bladder cancer to be associated with liquor consumption (Wynder & Goldsmith, 1977) this finding is probably spurious.

The positive association between brain-cancer incidence and liquor consumption was also markedly reduced on eliminating one extreme data-point. This positive relationship could also be dismissed as spurious, if it were not for the results of a recent cohort study of 4401 alcoholic World War II veterans (Robinette *et al.*, 1979). Although there were only 5 cases of brain cancer in the cohort, this was significantly in excess of the number expected. However, an earlier case-control study of central-nervous-system neoplasma actually found more consumers of alcohol (type unspecified) among the controls than the cases (Choi *et al.*, 1970). Clearly further studies on this question are needed.

In summary, the findings of this study are in agreement with the many previously described associations between cigarette smoking and cancer, and in addition, suggest several hypotheses which should be tested by well-designed case-control and cohort studies. Of particular interest was the association of beer consumption with cancer of the oesophagus, stomach, pancreas, lung and kidney. It is suggested that these association could be the result of the contamination of beers with the carcinogen dimethylnitrosamine, and that future epidemiological studies should attempt not only to collect information on consumption of different types of alcoholic beverages, but also to determine the favourite brands consumed and their DMN content. Although all alcoholic beverages contain alcohol, they are otherwise very dissimilar. Thus, it may well be necessary to distinguish between beverages and brands before consistent epidemiological associations will be found.

As previously discussed, there are many difficulties in interpretation of findings from correlation studies using populations as sampling units. Our findings should not be overinterpreted, but construed primarily as hypothesis-generating. In studies of the aetiology of human cancer, only data on exposure and outcome in the same individuals can be strong indications of a causal relationship.

This research was funded by Contract No. N01-CP-53511 from the U.S. National Cancer Institute, DHEW, and a grant from the United States Brewers Association.

We gratefully acknowledge the cooperation of Dr Thomas Burch of the Hawaii State Department of Health and Dr Will Rellahan of the Hawaii Tumor Registry and the technical assistance of Marlene Ng and Colleen Uyechi.

### REFERENCES

ANDERSON, L. M., PRIEST, L. J. & BUDINGER, J. M. (1979) Lung tumorigenesis in mice after chronic exposure in early life to a low dose of dimethylnitrosamine. *J. Natl Cancer Inst.*, **62**, 1553.

ARMSTRONG, B. & DOLL, R. (1975) Environmental factors and cancer incidence and mortality in different countries, with special reference to dietary practices. *Int. J. Cancer*, **15**, 617.

BRESLOW, N. E. & ENSTROM, J. E. (1974) Geographic correlation between cancer mortality rates and alcohol-tobacco consumption in the United States. *J. Natl Cancer Inst.*, **53**, 631.

BURCH, G. E. & ANSARI, A. (1968) Chronic alcoholism and carcinoma of the pancreas. *Arch. Int. Med.*, **122**, 273.

CHAN, W. C., COLBOURNE, M. J., FUNG, S. C. & HO, H. C. (1979) Bronchial cancer in Hong Kong 1976-1977. *Br. J. Cancer*, **39**, 182.

CHOI, N. W., SCHUMAN, L. M. & GULLEN, W. H. (1970) Epidemiology of primary central nervous system neoplasms. II: Case-control study. *Am. J. Epidemiol.*, **91**, 467.

DOLL, R. & PETO, R. (1976) Mortality in relation to smoking: 20 years' observation on male British doctors. *Br. Med. J.*, ii, 1525.

FRAUMENI, J. F. & MASON, T. J. (1974) Cancer mortality among Chinese Americans, 1950-69. *J. Natl Cancer Inst.*, **52**, 659.

FUJITA, M., TAKAMI, M., USUGANE, N., NAMPEI, S. & TAGUCHI, T. (1979) Enhancement of gastric carcinogenesis in dogs given N-methyl-N-nitro-N-nitrosoguanidine following vagotomy. *Cancer Res.*, **39**, 811.

GOFF, E. U. & FINE, D. H. (1979) Analysis of volatile N-nitrosamines in alcoholic beverages. *Fd Cosmet. Toxicol.*, **00**, 000.

HAENSZEL, W. & KURIHARA, M. (1968) Studies of Japanese migrants: I. Mortality from cancer and other diseases among Japanese in the United States. *J. Natl Cancer Inst.*, **40**, 43.

HAENSZEL, W., KURIHARA, M., SEGI, M. & LEE, R. K. C. (1972) Stomach cancer among Japanese in Hawaii. *J. Natl Cancer Inst.*, **49**, 969.

ISHII, K., NAKAMURA, K. & OZAKI, H. (1968) Problems in the epidemiology of pancreas cancer. *Jap. J. Clin. Med.*, **26**, 1839.

JENSEN, O. M. (1979) Cancer morbidity and causes of death among Danish brewery workers. *Int. J. Cancer*, **23**, 454.

KOLONEL, L. N., HIROHATA, T. & NOMURA, A. M. Y. (1977) Adequacy of survey data collected from substitute respondents. *Am. J. Epidemiol.*, **106**, 476.

KONO, S. & IKEDA, M. (1979) Correlation between cancer mortality and alcoholic beverage in Japan. *Br. J. Cancer*, **40**, 449.

KREYBERG, L. (1962) Histological lung cancer types: A microbiological and biological correlation. *Acta Pathol. Microbiol. Scand. Suppl.*, **157**, 19.

MACLENNAN, R., DACOSTA, J., DAY, N. E., LAW, C. H., NG, Y. K. & SHAUMUGARATNAM, K. (1977) Risk factors for lung cancer in Singapore Chinese, a population with high female incidence rates. *Int. J. Cancer*, **20**, 854.

MARTINEZ, I. (1969) Factors associated with cancer of the esophagus, mouth and pharynx in Puerto Rico. *J. Natl Cancer Inst.*, **42**, 1069.

MEYER, F. (1977) Relations alimentation-cancer en France. *Gastroenterol. Clin. Biol.*, **1**, 971.

MODAN, B., BARELL, V., LUBIN, F., MODAN, M., GREENBERG, R. A. & GRAHAM, S. (1975) Low-fiber intake as an etiologic factor in cancer of the colon. *J. Natl Cancer Inst.*, **55**, 15.

NAPALKOV, N. P. & POZHARISSKI, K. M. (1969) Morphogenesis of experimental tumors of the esophagus. *J. Natl Cancer Inst.*, **42**, 922.

POUR, P., ALTHOFF, J., KURGER, F. W. & MOHR, U. (1977) A potent pancreatic carcinogen in Syrian hamsters: N-nitrosobis (2-oxo-propyl) amine. *J. Natl Cancer Inst.*, **58**, 1449.

RELLAHAN, W., RASHAD, M. N., BURCH, T. A. & OKINAGA, N. S. G. (1975) *Cancer Patterns in Hawaii.* Honolulu: The Cancer Center of Hawaii and Hawaii State Department of Health.

ROBINETTE, C. D., HRUBEC, Z. & FRAUMENI, J. F. (1979) Chronic alcoholism and subsequent mortality in World War II veterans. *Am. J. Epidemiol.*, **109**, 687.

ROTHMAN, K. (1975) Alcohol. In *Persons at High Risk of Cancer.* Ed. Fraumeni. New York: Academic Press. p. 139.

ROTHMAN, K. & KELLER, A. (1972) The effect of joint exposure to alcohol and tobacco on risk of cancer of the mouth and pharynx. *J. Chronic Dis.*, **25**, 711.

SCHRAUZER, G. N. (1976) Cancer mortality correlation studies. II. Regional associations of mortalities with the consumption of foods and other commodities. *Med. Hypotheses*, **2**, 39.

SPIEGELHALDER, B., EISENBRAND, G. & PREUSSMAN, R. (1979) Contamination of beer with trace quantities of N-nitrosodimethylamine. *Fd Cosmet. Toxicol.*, **17**, 29.

STOCKS, P. (1970) Cancer mortality in relation to national consumption of cigarettes, solid fuel, tea and coffee. *Br. J. Cancer*, **24**, 215.

TERRACINI, B., PALESTRO, G., GIGLIARDI, M. R. & MONTESANO, R. (1966) Carcinogenicity of dimethylnitrosamine in Swiss mice. *Br. J. Cancer*, **20**, 871.

U.S. DEPARTMENT OF HEALTH, EDUCATION AND WELFARE (1979) *Smoking and Health—a report of the Surgeon General.* Washington: U.S. Government Printing Office.

WILLIAMS, R. R. & HORM, J. W. (1977) Association of cancer sites with tobacco and alcohol consumption and socioeconomic status of patients: Interview study from the Third National Cancer Survey. *J. Natl Cancer Inst.*, **58**, 525.

WORLD HEALTH ORGANIZATION (1976) *International Classification of Diseases for Oncology.* Geneva. p. 1.

WYNDER, E. L. & BROSS, I. J. (1961) A study of etiological factors in cancer of the esophagus. *Cancer*, **14**, 389.

WYNDER, E. L., COVEY, L. S., MABUCHI, L. & MUCHINSKI, M. (1976) Environmental factors in cancer of the larynx. A second look. *Cancer*, **38**, 1951.

WYNDER, E. L. & GOLDSMITH, R. (1977) The epidemiology of bladder cancer. *Cancer*, **40**, 1246.

WYNDER, E. L., MABUCHI, K., MARUCHI, N. & FORTNER, J. G. (1973) Epidemiology of cancer of the pancreas. *J. Natl Cancer Inst.*, **50**, 645.

WYNDER, E. L., MABUCHI, K. & WHITMORE, W. F. (1974) Epidemiology of adenocarcinoma of the kidney. *J. Natl Cancer Inst.*, **53**, 1619.

ZAK, F. G., HOLZNER, J. H., SINGER, E. J. & POPPER, H. (1969) Renal and pulmonary tumors in rats fed dimethylnitrosamine. *Cancer Res.*, **20**, 96.